# Genome-wide analysis of single-locus and epistasis single-nucleotide polymorphism effects on anti-cyclic citrullinated peptide as a measure of rheumatoid arthritis

**DOI:** 10.1186/1753-6561-1-s1-s127

**Published:** 2007-12-18

**Authors:** Li Ma, Daniel Dvorkin, John R Garbe, Yang Da

**Affiliations:** 1 Department of Animal Science, University of Minnesota, 1364 Eckles Avenue, St. Paul, Minnesota 55108, USA

## Abstract

The goal of this study was to identify single-locus and epistasis effects of SNP markers on anti-cyclic citrullinated peptide (anti-CCP) that is associated with rheumatoid arthritis, using the North American Rheumatoid Arthritis Consortium data. A square root transformation of the phenotypic values of anti-CCP with sex, smoking status, and a selected subset of 20 single-nucleotide polymorphism (SNP) markers in the model achieved residual normality (*p *> 0.05). Three single-locus effects of two SNPs were significant (*p *< 10^-4^). The epistasis analysis tested five effects of each pair of SNPs, the two-locus interaction, additive × additive, additive × dominance, dominance × additive, and dominance × dominance effects. A total of ten epistasis effects of eight pairs of SNPs on 11 autosomes and the X chromosome had significant epistasis effects (*p *< 10^-7^). Three of these epistasis effects reached significance levels of *p *< 10^-8^, *p *< 10^-9^, and *p *< 10^-10^, respectively. Two potential SNP epistasis networks were identified. The results indicate that the genetic factors underlying anti-CCP may include single-gene action and gene interactions and that the gene-interaction mechanism underlying anti-CCP could be a complex mechanism involving pairwise epistasis effects and multiple SNPs.

## Background

The data set of the North American Rheumatoid Arthritis Consortium (NARAC) for Genetic Analysis Workshop 15 (GAW15) contains genotypes of 5700 SNPs covering all 23 human chromosomes, affected status of rheumatoid arthritis (RA), and a number of quantitative traits including anti-cyclic citrullinated peptide (anti-CCP). Anti-CCP is associated with RA and is used by some as a measure of RA [1]. Linkage analysis of the RA status in the NARAC data using affected sib-pair method has been reported [2].

## Methods

**Figure 1:**
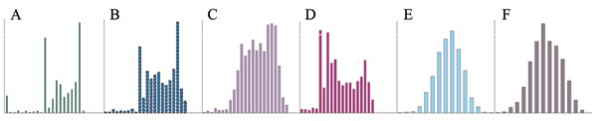
**Histograms of anti-CCP distributions**. A-D significantly deviated from the normal distribution (*p *< 0.01). The deviations from normal distribution were insignificant in E (*p *> 0.15) and F (*p *> 0.05). A, Phenotypic distribution of anti-CCP values; B, residual distribution of anti-CCP values under the model with sex and smoking status as the fixed effects; C, residual distribution of anti-CCP values under the model with sex, smoking status, and all 37 SNPs with significant single-locus and epistasis effects as the fixed effects; D, residual distribution of the square root transformed anti-CCP values under the model with sex and smoking status as the fixed effects; E, residual distribution of the square root transformed anti-CCP values under the model with sex, smoking status, and all 41 SNPs with significant single-locus and epistasis effects as the fixed effects; F, residual distribution of the square root transformed anti-CCP values under the model with sex, smoking status, and a minimal subset of 20 SNPs from the 41 SNPs in E, to achieve normality.

The NARAC data set was edited by requiring each individual to have SNP genotypes on the 5700 SNPs and anti-CCP record, and 1466 individuals satisfied this criterion. The anti-CCP values significantly deviated from normal distribution with *p *< 0.01 (Fig. 1A), according to the Shapiro-Wilk, Kolmogorov-Smirnov, Cramer-von Mises, and Anderson-Darling tests offered by SAS UNIVARIATE PROCEDURE [3]. Because the residual normal distribution, not the phenotypic normal distribution, is required for the statistical tests in this article, a statistical model that achieves residual normality was found using the procedure leading to Figures 1B–1F. The untransformed anti-CCP did not achieve residual normality (*p *< 0.01; Fig. 1B–1C). The Box-Cox transformation [4] for a range of λ values and the square root transformation of anti-CCP were evaluated to find an optimal transformation that has the minimal sum of squares under the model for Figure 1B and improves the residual normality. None of the transformations achieved residual normality (*p *< 0.01), but the square root transformation was found to have minimal residual sum of squares. The residual distribution under this transformation is shown in Figure 1D. For the model used in Figure 1D, a total of 41 SNPs were found to have significant single-locus and epistasis effects with the same significance level as the SNPs for Figure 1C. Adding all the 41 SNPs in the model for Figure 1D achieved a near-perfect residual normality (*p *> 0.15; Fig. 1E). To reduce the model degrees of freedom or increase the residual degrees of freedom, step-wise elimination of SNPs from the full model for Figure 1E was conducted to find the minimal set of SNPs that had 20 SNPs (Table 1) and still achieved residual normality for the transformed data (*p *> 0.05; Fig. 1F). In the model for Figure 1F, each SNP with a single-locus effect was fitted in model as a locus with three genotypes while each pair of SNPs were fitted in the model as a genetic factor with nine (3 × 3) genotypes. Each SNP or SNP pair in this subset was re-tested by treating the other SNPs in the set as fixed effects (in addition to sex and smoking status). For all SNPs not in this subset, the SNP effects were tested based on the model for Figure 1F. The model for testing single-locus effects was

(*y*)^1/2 ^= *sex *+ *CigE *+ (*normality SNPs*) + *SNP *+ *e*,

**Table 1:** Minimal set of SNPs to achieve residual normality of the square root transformed anti-CCP values in Figure 1F

Chromosome 2	SNP1	Chromosome 2	SNP2
2	rs2685263	-^a^	-
6	rs1892512	-	-
2	rs289847	16	rs9033
2	rs981395	23	rs1108444
3	rs13975	5	rs1016256
6	rs1398576	18	rs884205
8	rs1000236	9	rs751340
12	rs1400142	14	rs1959068
12	rs1843910	22	rs743409
14	rs1033594	17	rs759563
16	rs194790	17	rs1872076

^a^-, The left marker had a significant single-locus effect.

where (*y*)^1/2 ^is the square root transformed anti-CCP, *sex *is the gender of the individual, *CigE *is the indicator variable whether the person ever smoked, (*normality SNPs*) is the 20 SNPs in Table 1 to achieve residual normality shown in Figure 1F, *SNP *is the SNP being tested for three single-locus effects (the SNP marker effect, and additive and dominance effects), and *e *is the random residual. The significance test of the SNP marker effect used an *F*-test, and *t*-tests were used to test additive and dominance effects. The epistasis analysis tested five effects of each pair of SNPs: two-locus interaction (I), additive × additive (A × A), additive × dominance (A × D), dominance × additive (D × A), and dominance × dominance (D × D) epistasis effects. The genetic interpretation of the A × A, A × D, D × A, and D × D epistasis effects are allele × allele, allele × genotype, genotype × allele, and genotype × genotype interactions, respectively. The model for testing epistasis effects was

(*y*)^1/2 ^= *sex *+ *CigE *+ (*normality SNPs*) + *SNP1 *+ *SNP2 *+ SNP1*SNP2 + *e*,

where SNP1*SNP2 is the interaction effect between the two SNPs, to be denoted by "*I*". The significance test of the *I*-effect used an *F*-test, and *t*-tests were used to test four individual epistasis effects, A × A, A × D, D × A, and D × D, using an extended Kempthorne model that allows Hardy-Weinberg and linkage disequilibria [5]. The *I*-effect answers the question whether the two loci had an interaction whereas an individual epistasis effect identifies the exact mode of the interaction. For testing epistasis effects involving the X chromosome, only females were included in the analysis. The single-locus and epistasis tests using Models (1–2) were implemented using the epiSNP computer package developed by the authors [6].

## Results

### Significant single-locus SNP effects

The single-locus analysis using Model (1) had 17,100 tests for 5700 SNPs. Two SNPs on chromosomes 2 and 3 had three significant effects on the transformed data with *p *< 10^-4 ^(Table 2). With the stringent significance level of *p *< 10^-4^, 17,100 tests are expected to yield 1.7 significant results by chance. Therefore, the three significant effects of two SNPs could be due to chance. Results with less stringent significance levels may be of interest to compare with reported results for possible confirmations. A total of 231 single-locus effects of all chromosomes were significant at *p *< 0.01.

**Table 2:** Significant single-locus SNP effects on square root transformed anti-CCP with *p *< 10^-4^

Chromosome	SNP	Chromosome location (bp)	Effect	*p*-Value
2	rs1017267	539,873	Marker	0.525 × 10^-4^
2	rs1017267	539,873	Dominance	0.403 × 10^-4^
3	rs1401337	149,511,657	Marker	0.628 × 10^-4^

### Significant epistasis effects

The epistasis analysis using Model (2) had 81,210,750 tests for 5700 SNPs. Ten significant epistasis effects of eight pairs of SNPs on 11 autosomes and the X chromosome reached the significance level of *p *< 10^-7 ^(Table 3). Of these ten effects, three effects of two pairs of SNPs reached significance levels of *p *< 10^-8^, *p *< 10^-9^, and *p *< 10^-10^, respectively (Table 3). The most significant epistasis effect was an additive × additive effect between two SNPs on chromosome 11 and the X chromosome, followed by a dominance × additive effect between chromosomes 7 and 10. With the significance level of *p *< 10^-7^, the expected number of significant results out of 81,210,750 tests due to chance is eight, so that ten observed significant epistasis effects were more than expected by chance. More importantly, the three significant epistasis effects with *p *< 10^-8^, *p *< 10^-9^, and *p *< 10^-10 ^far exceeded the significance level defined by the threshold *p*-value of 10^-7 ^to be considered as random results. Based on this analysis, evidence for epistasis effects was stronger than single effects reported in this article.

**Table 3:** Significant SNP epistasis effects on square root transformed anti-CCP with *p *< 10^-7^

Chromosome 1	SNP1	Chromosome 2	SNP2	Effect^a^	*p*-Value
1	rs2031750	18	rs1395610	A × A	0.827 × 10^-7^
2	rs1261238	23	rs953114	D × A	0.976 × 10^-7^
					
6	rs310389	13	rs4941527	I	0.563 × 10^-7^
6	rs310389	13	rs4941527	D × A	0.765 × 10^-7^
					
6	rs873460	16	rs874562	A × A	0.754 × 10^-7^
6	rs1087924	23	rs644429	I	0.583 × 10^-7^
7	rs2056553	10	rs1328327	D × A	0.583 × 10^-8^
					
11	rs1009172	23	rs17410	I	0.612 × 10^-9^
11	rs1009172	23	rs17410	A × A	0.247 × 10^-10^
12	rs1400142	14	rs1959068	D × D	0.437 × 10^-7^

^a^I, two-locus interaction effect; A × A, additive × additive effect; A × D, additive × dominance effect; D × A, dominance × additive effect; D × D, dominance × dominance effect.

### Complex gene interaction mechanism and epistasis network

**Figure 2:**
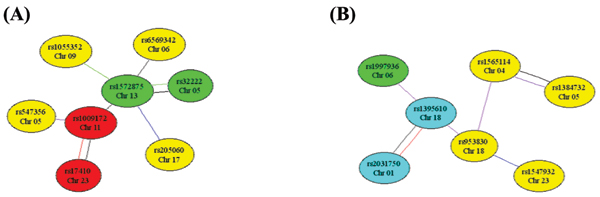
**Two potential epistasis networks each centered at an SNP that had one of the most significant epistasis effects and interacted with other loci at lower significance levels**. Each type of epistasis effect is represented by the line color: black, I effect; red, A × A; purple, A × D; blue, D × A; green, D × D. A pair of SNPs connected by two lines had two significant epistasis effects. The significance level of each epistasis effect is represented by the node color: red, *p *< 10^-10^; cyan, *p *< 10^-7^; green, *p *< 10^-6^; yellow, *p *< 10^-5^. A, Epistasis network centered at rs1009172 of chromosome 11; B, Epistasis network centered at rs1395610 of chromosome 18.

The epistasis results indicate that the gene interaction mechanism underlying anti-CCP could be a complex mechanism involving three types of epistasis effects and multiple SNPs, where 'three types of epistasis' refers to A × A, A × D or D × A, and D × D. The exact content of this complex mechanism can be illustrated using the example of three highly significant pairs of SNPs, rs1009172 (chromosome 11) and rs17410 (chromosome X), rs2056553 (chromosome 7) and rs1328327 (chromosome 10), and rs1400142 (chromosome 12) and rs1959068 (chromosome 14). The exact gene interaction mechanism of these three pairs is (A × A)-(D × A)-(D × D). Close examination of each of the three epistasis effects showed that individuals with *1_1 *allele combination of rs1009172 (chromosome 11) and rs17410 (chromosome X) SNP pair, *22_2 *genotype-allele combination of rs2056553 (chromosome 7) and rs1328327 (chromosome 10) SNP pair, and *11_12 *genotype-genotype combination of rs1400142 (chromosome 12) and rs1959068 (chromosome 14) SNP pair had the lowest square root transformed anti-CCP values. In contrast, individuals with *1*_*2 *allele combination of rs1009172 (chromosome 11) and rs17410 (chromosome X) SNP pair, *12_2 *genotype-allele combination of rs2056553 (chromosome 7) and rs1328327 (chromosome 10) SNP pair, and *11_11 *genotype-genotype combination of rs1400142 (chromosome 12) and rs1959068 (chromosome 14) SNP pair had the highest square root transformed anti-CCP values. In addition to highly significant epistasis effects, an epistasis network around a locus that has a highly significant epistasis effect with at least one locus and interacted with a large number of other loci at a lower significance level (higher *p*-value) should be of interest, because many significant epistasis effects involving one locus at a lower significance level are less likely to be random than a single epistasis effect at the same significance level. Figure 2 shows two examples of such epistasis network.

## Discussion

The results showed that evidence for epistasis effects was stronger than for single-locus effects on anti-CCP. This implies that gene interactions could be an important genetic factor underlying rheumatoid arthritis. The phenotypic distribution of anti-CCP might merit further study to identify factors that caused the distribution curve with multiple peaks as shown in Figure 1A. Including such factors in the statistical model could achieve residual normality without using as many 'normality SNPs' and hence increases the residual degrees of freedom.

## Conclusion

The genetic factors underlying anti-CCP may include single-gene action and gene interactions but gene interaction effects could be more important than single-gene effects. The gene interaction mechanism could be a complex mechanism involving a number of SNPs and three types of pairwise epistasis effects, and the epistasis results could be used to identify allelic and genotypic combinations with the highest and lowest anti-CCP levels.

## Competing interests

The author(s) declare that they have no competing interests.
